# Long term evolution of patients treated in a TIA unit

**DOI:** 10.1186/1755-7682-6-19

**Published:** 2013-05-01

**Authors:** Lorena Benavente, Sergio Calleja, Davinia Larrosa, Juan Vega, Gerard Mauri, Julio Pascual, Carlos H Lahoz

**Affiliations:** 1 Neurology Department, University Hospital “Central de Asturias”, C/Julián Clavería s/n, 33006, Oviedo, Spain

**Keywords:** TIA, Mild stroke, TIA unit, Stroke care models, TIA management

## Abstract

**Background:**

Transient ischemic attacks (TIA) entail a high risk of stroke recurrence, which depends on the etiology. New organizational models have been created, but there is not much information about the long-term evolution of patients managed according to these premises. Our aim is to refer the follow-up of patients attended according to our model of TIA Unit.

**Methods:**

TIA Unit is located in the Emergency Department and staffed by vascular neurologists. Patients admitted during the Neurology night shift stayed in such Unit <48h with complete etiological study. Preventive treatment is instituted in patients discharged to a high resolution Neurology consult, in order to review in <2 weeks and subsequent follow-up.

**Results:**

During a year 161 patients were attended, being admitted to the hospital 8.6%. A total of 1470 hospital days were avoided. Recurrence at 90 days was of 0.6%. Mean follow-up was 18.14 ± 8.02 months (0–34), total recurrence 6.2% (70% cardioembolic strokes). There were no complications derived from treatment. Cardiological events were recorded in 10.6%, neoplastic in 5%, cognitive impairment in 11%. There were 3 deaths unrelated nor to the stroke or its treatment.

**Conclusions:**

This model allows an early diagnosis and treatment of TIA, preventing recurrences of stroke in a long term. It detects atherothrombotic strokes, most of them admitted to the hospital, and it shows a greater difficulty for detecting all cardioembolic strokes. TIA Unit appeared to be safe in using anticoagulation therapy, as the follow-up shows. It shows the same quality of management than hospital admission, with a significant saving in hospital stays.

## Background

The threat that transient ischemic attack represents for suffering a stroke is well known and was stated many decades ago. However, the pessimism that was classically a part of the management in the treatment of strokes also affects TIA by proximity. The importance of time in saving the brain was even less present when the clinical presentation was ephemeral, without any squeal, transient and reversible, as TIAs are.

Aspects of the physiopathology and management of TIAs were published in the 1970’s [1,2]. Literature states the adequate treatment of TIAs as this one which goes through the corresponding aetiology. It is over the past few years when TIAs are started to be given a more objective importance, consistently with the risk that they represent. They are considered the same illness as the established strokes, which is cerebral ischemic disease.

The adequate treatments, derived from a concept of the aetiology of TIAs or minor stroke, come to reduce the risk of recurring stroke by 80–90% [3]. Over the past few years, attempts have been made to establish different strategies for improving the management of TIA and making it effective and safe [4] in the prevention of stroke. In this sense, TIA Units arise with different study models, which are the reason why we propose a model adapted to our healthcare reality, and we analyse its effectiveness and safety.

## Methods

**Figure 1:**
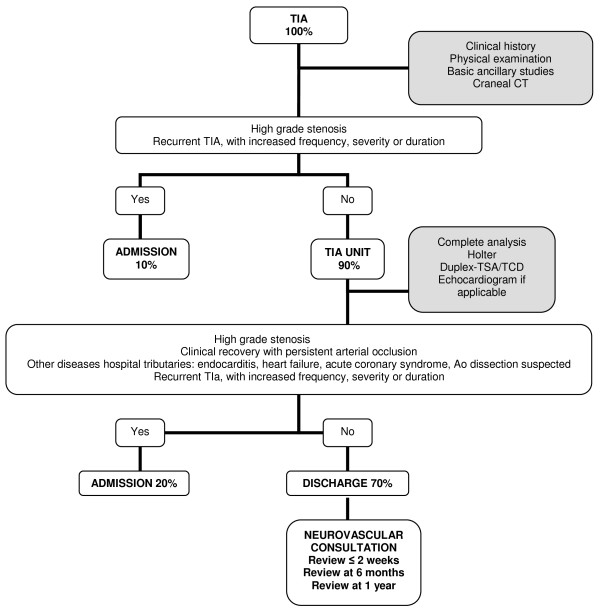
Criteria to admission in TIA Unit or Neurology room.

The TIA Unit is located in the Emergency Department of the Central University Hospital of Asturias, which covers a population of 342,020 inhabitants [5]. It is a hospital with a Neurology department on a 24-hour shift, where the clinical presentation suggesting a TIA is assessed at the request of the Urgent care physician. Patients with compatible signs and symptoms, according to the existing recommendations [6-9], are finally admitted in this TIA Unit. Its aim is to reduce hospital admissions by 70%. Patients who fill the admission criteria (see Figure 1) stay in TIA Unit after a complete blood count, coagulation tests, cardiac markers, a basic chemistry panel, a 12-lead ECG, a chest x-ray and a brain CAT scan have been carried out. Until the etiologic study is practised, treatment is iniciated with liquid infusion, low-molecular-weight heparins at a dose of 0.1 mL/10 kg of weight/d, antiplatelet therapy with 100 mg of acetyl salicylic acid, and 5 milligrams of enalapril in case the blood pressure is ≥220/120 mmHg, quite similar to any stroke of unknown etiology. A Holter is placed on all the patients without a known cardioembolic disease, which is reported by Cardiology the next day. The following morning, the chemistry panel is completed with a lipid profile, liver function, proteins, thyroid function, vitamins, C-reactive protein and homocysteine. If the patient is <55, the analysis of autoantibodies and blood serologies for syphilis, Borrelia, Brucella and neurotropic viruses is also carried out. A vascular neurologist assesses the patient once again and carries out a Duplex of supra-aortic trunks, as well as a transcranial Doppler ultrasound. In specific cases, according to criteria shown in Table 1, a transthoracic or transoesophageal ultrasound will also be carried out. And this study can be postponed for a maximum of one week, in coordination with the Cardiology Department. Patients are discharged with a report and preventive treatment, referring them to a new Neurovascular consult within a time frame of less than 15 days, where the treatment is adjusted in terms of all the complementary studies, and where a follow-up is practiced at 6 months and one year.

**Table 1:** Indications for echocardiography

** Indications for echocardiography**	
Multiple areas	Unless known embolic source
<55 year-old	Unless evident cases of non-cardioembolic aetiology
Undetermined TIA	Non-lacunar with non-relevant cervical duplex and TCD, sinus rhythm and unknown cardioembolic source
Suspected cardioembolic source	Heart murmur, heart failure, typical chest pain, altered ECG despite sinus rhythm
Known cardioembolic source susceptible from therapy changes	Valvular prosthesis, suspected endocarditis, ischemic heart disease or dilated cardiomiopathy for which the last echocardiography was carried out over 6 months prior to the study.

Demographic data, studies results, treatments and recurrences and comorbidity that occurred during the follow-up were recorded and analysed as a descriptive statistical study according to the free distribution software R.2.10 (http://www.r-project.org). This is an observational study from the clinical practise, but the Ethics Committee from the hospital approved the use of data.

## Results

Between August, 2008, and July, 2009, 161 patients were seen at the TIA Unit of the HUCA, 14 of which required an admission to the hospitalization ward (8.6%), a figure that is far below the 30% aimed with the study design. 75.7% of the cases were >65 years-old, with an overall mean age of 81.1. The delay in care is very significant, with a mean of 5.1 hours and a mean duration of the symptoms of less than 7.02 hours. This contrasts with the usual duration of true TIAs (30–60 minutes), revealing the banal nature that is still being attributed to this entity. Regarding the complementary studies, the ultrasound detected 14.7% of intracranial or extracranial vascular stenosis that were predominantly asymptomatic. The Holter electrocardiographic recording was pathological in 42% of the patients, of which 33.6% were atrial fibrillations. Advanced atrioventricular blocks and severe pause led to the immediate placement of pacemakers. Echocardiography, carried out in 37 patients, 8 of which were transoesophageal, was relevant from a pathological standpoint for the aetiology of the stroke in 14 patients (37.8%). The treatments at discharge from the TIA Unit were mainly LMWH (53.5%), because it is frequent to carry out the Holter lecture in the subsequent days. So we maintain LMWH until then and we adjust definite treatment with oral anticoagulation or keep platelets at the Neurology consult within the following 10 days, at most. Antiplatelet therapy was present in 46.5%; statins in 10%, a very low percentage, in which many cases the analytical results were pending, also to be adjusted at the consult. In 3.7% of the cases, antihypertensive drugs were newly indicated to leave blood pressure figures at the hands of the Primary Care physician once the acute phase of the stroke had been overcome. All of these characteristics are shown in detail in Table 2.

**Table 2:** General results

**Admission (8.6%)**	**Topographical diagnosis**	**Aetiological diagnosis (toast) **^**10**^	**Clinical presentation**	**US Stenosis**	**Holter**	**TTE/TEE**	**Treat**
Persistence of clinical signs and symptoms 1	Left H. 42%	Cardioembolic 47%	Dysphasia 13% Monoparesis 11.2%	BA 4.3%	AF 36	Atypical akinesia 5	LMWH 53.5%
Clinical instability due to comorbidity 3	Right H. 23%	Cryptogenic 35%	Facial paresis 8.1%	ICA 3.7%	AT 2	PFO 4	ASA 29.2%
Symptomatic cervical stenosis 6	V-B 18%	Atherothrombotic 7%	Dysarthria 8.1% Hemiparesis 6.2%	VA 3.1%	advanced AVB 2	Severe MI with dilated LA 2	clopidogrel 9.6%
Symptomatic intracranial stenosis 3	Imprecise 17%	Lacunar 5%	Ataxia 3.1% Diplopia 1.8% Hemianopsia 0.6%	MCA 1.8%	ABV-II 2 USVT 2	EF < 30% 1	triflusal 7.7% statins 10%
		Undetermined due to several causes 5%		ACA 1.2%	Severe pause 1	IAC 1	ACEI 1.2% ARA-II 2.5%
				PCA 0.6%		AF 1	

[10]: Number of reference of TOAST classification in the manuscript.

Considering an average hospital stay of 9.95 days for admitted TIAs, and taking into account the 147 days avoided in the first year, a figure of 1470 hospital stays have been saved.

Recurrence at 90 days was of 0.6%. One patient suffered a stroke at 24 hours while following the treatment with LMWH, ASA 100 mg and atorvastatin 20 mg, pending the reading of the Holter recording, which turned out to be normal. The TSA ultrasound showed non-significant bilateral atheromatosis.

**Figure 2:**
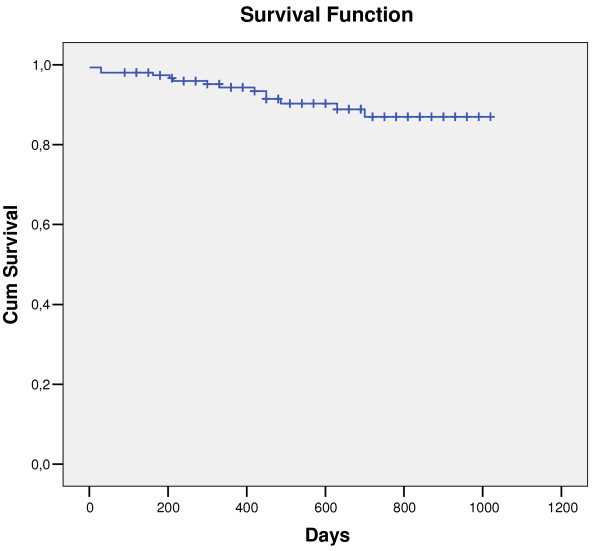
**Survival curve for recurrence or death.** Kaplan-Meier curve.

The mean follow-up was of 18.14 ± 8.02 months (0–34) and the total recurrence during all that time was of 6.2%. 30% of the patients who recurred were of cryptogenic aetiology and continued being thus after repeating blood tests, a 24-hour Holter and the echocardiography. Another 70% was of cardioembolic TIAs: 40% of these recurred in spite of being correctly anticoagulated (INR >2), but 30% had previously been cryptogenic and, after the recurrence, the new Holter registry showed atrial fibrillation. The details of the recurrences and the survival curve for recurrences or death are reflected in Table 3 and in Figure 2.

**Table 3:** Characteristics of the recurrences

	
1	TIA at 7 months: Negative studies again; TIA at 10 months with paroxysmal atrial fibrillation in the third Holter
2	Acute coronary syndrome at 1 month; TIA at 5 months with paroxysmal atrial fibrillation in the second Holter
3	TIA vs. seizures (3 visits to the emergency room): low INR, associated levetirazetam
4	Stroke at 24 hours: previously negative studies; treatment: LMWH + ASA + atorvastatin
5	Vertebro-basilar stroke at 7 months; previously cardioembolic TIA (INR 5.32)
6	TIA at 16 months with paroxysmal atrial fibrillation in the second Holter
7	Stroke at 10 months (PFO on antiplatelet therapy, anticoagulation treatment is started)
8	Stroke at 15 months (PFO on antiplatelet therapy, anticoagulation treatment is started)
9	Stroke at 23 months; previously cardioembolic TIA (INR 3.28)

The mortality at this time was of 1.8%, comprised by three patients. A patient died 10 days after a cardioembolic TIA; the patient was still not anticoagulated and suffered an acute pancreatitis and a frontotemporal cerebral haematoma. Another patient was a diagnostic error of minor stroke, due to ptosis, showing a meningeal carcinomatosis at 7 months and dying from a pulmonary thromboembolism. The other patient died due to a gastrointestinal bleeding when placing a percutaneous endoscopic gastrostomy, after suspecting a multisystem atrophy one year later. Cardiological comorbidity was also recorded in 10.6%, neoplastic comorbidity in 5% or neurological comorbidity, whose characteristics are reflected in Table 4.

**Table 4:** Comorbidity

**Cardiological**	**Neoplastic**	**Neurological**
Cardiology admission at 24 h:	1 pancreatic carcinoma with liver metastases secondary to liver transplantation for HCV cirrhosis	2 patients with suspected seizures
• 2 pacemaker placements (slow atrial fibrillation; advanced AV block)		
		1 patient with a real seizure few months after the TIA
• Acute coronary syndrome		
Orthostatic syncope later admitted in Cardiology	1 carcinoma i.s. of vocal cord	1 patient with falls (parkinsonism)
Recurrent syncope (subcutaneous Holter)	2 colon carcinomas	1 optic neuropathy (fistula)
Angina consultation: 2 patients	1 pelvic cystic tumour	1 head trauma in an anticoagulated patient without incidences
Heart failure: 7 patients with income, some multiple	1 Non-Hodgkin’s mantle-cell lymphoma in amygdala	1 ventriculoperitoneal shunt for hydrocephalus
Prosthetic valves: 2 patients	1 prostate carcinoma	1 Wernicke encephalopathy
Anaemia and heart failure: 1 patient; discontinued anticoagulants	1 lung cancer	10 patients (6.2%) with a diagnosis of cognitive impairment during follow-up, mean age 81.1 years
	1 pulmonary nodule study	

If we excluded possible mixed cases, those with some cardiological or neurological comorbidity and perhaps TIA mimics (2 patients with placement of pacemaker; 1 orthostatic syncope; 1 recurrent syncope; 2 pathologic valves; 2 suspected seizures; 1 real seizure a few months later; 1 patient with recurrent extrapyramidal falls; 1 optic neuropathy secondary to a fistula; 1 Wernicke encephalopathy), recurrence at 90 days would keep on 0.67%, and mortality 1.3% at the end of the follow-up.

## Discussion

TIAs precede more than one fourth of established strokes [11]. Symptoms of TIA are frequently ignored by the patients and their relatives, or misdiagnosed by physicians, which delays the diagnosis and treatment. Moreover, when the patient is admitted, his enrolling in the hospital organization involves times that are more or less extended for carrying out all the complementary studies. As for patients who come in to Urgent Care with symptoms that are compatible with TIA, it is usual for them to be sent back to their Primary Care physician, who in the best-case scenario, refers them for a further outpatient study by Neurology [12,13], with the consequential delay in the diagnosis and optimal treatment. Taking into consideration that the risk of stroke after suffering a TIA is up to 15% in the first 15 days [14-16], the ineffectiveness of the current management of TIA is easily deduced, as it is the impossibility derived to prevent strokes.

There are no randomized trials with results that mark a universal guideline to follow of the appropriate care of TIA. The advance of the different studies published about this disease leads to a common conclusion that involves its early management, with the ensuing aetiological study and consequent treatment. The scenario can be very different depending on the country, as it can vary between the different regions of a same country.

One of the healthcare realities that has studied most in terms of management of TIAs and minor stroke, and where the development of TIA units is probably most advanced, is the United Kingdom. Models designed in the EXPRESS study, a population-based study in the Oxford area that presents two different phases of intervention [17-19], are especially relevant. Another model of TIA Unit is the one developed in Paris, under the sponsorship of the SOS-TIA study [20].

Our alternative seems safe and effective, with a unit of short stay associated to the area of Urgent care, where patients with TIA can be admitted and undergo aetiological studies in the first 24–48 hours, starting the treatment immediately. The results of recurrence obtained after 90 days that were even better than those of the large reference studies (0.6% compared to 10.3% in the phase 1 and 2.1% in the phase 2 of the EXPRESS study 15 and 1.24% of the SOS-TIA study18).

In our series, within a maximum follow-up of almost 3 years, the recurrence rate was of 6.2%. However, we are not able to present a case-control study, because we don’t have previous values of recurrence in our hospital. But we know its natural history [1,2], and stroke recurrence at 3 months reaches 15–20%. In our hospital, TIA cases came to the Neurology ambulatory consultation some weeks after the symptoms appear. They came from the general physician or Emergency room. Those attended by neurologist in the Emergency room used to be admitted, which supposed a stay of more than 10 days for carrying out the etiologic study. Moreover, their aetiology is another notable point of discussion. Even if large-vessel atherothrombotic stroke is classically considered to have the greatest risk of recurrence [21], in our series, 70% of recurrences had a cardioembolic origin. 40% of the patients were in the therapeutic range of oral anticoagulation and 30% were of newly-diagnosed cardioembolic aetiology when the Holter recording was repeated. From this fact, the cost-effectiveness of repeating studies of electrocardiographic monitoring can be deduced, even though it is not an express indication in current clinical practice.

The agreement between different observers is sometimes an issue for the differential diagnosis of TIA. Multiple conditions may share symptoms or can even be superimposed semiologically to a TIA [22,23]. There are some characteristic symptoms that typically correlate with the vascular aetiology [24]. Moreover, the ABCD2 scale, which assesses age, blood pressure, clinical presentation, the duration of the symptoms and the presence of diabetes, also seems to correlate with a vascular aetiology when high scores are obtained [25]. However, there is always a percentage of patients incorrectly diagnosed with ischemic cerebral disease, ranging between 13 and 19%, depending on the series [26,27]. When the diagnosis is established after neuroimaging techniques and laboratory results, this percentage can fall down to 4% [28]. And if MRI techniques are also associated, the incidence falls to 1–2% [29]. In this way, our study is limited, as we were unable to carry out MRIs acutely in our patients. It is worth noticing some possible diagnostic errors of TIA, such as cardiological signs and symptoms, -especially of the syncope type detected in some cases-, as well as possible seizures, falls due to Parkinsonism, and fistulas involving the ocular globe, among others. In any case, the differential diagnosis of TIA depends greatly on the healthcare professional performance; and as they are transient symptoms, they make it more difficult to diagnose cerebral ischemia with certainty. Thus, taking into account all the patients sent to a TIA unit by general practitioners, and who were diagnosed by a neurologist expert in vascular disease, 65% have a confirmed diagnosis of TIA or minor stroke and 13% are diagnosed with a possible TIA, showing an error of 35% by general practitioners and a diagnostic doubt of 13% by neurovascular experts [18]. This percentage is similar in other series [30], and it is of 5% in our case.

Comorbidity is frequent in patients with TIA, as well as in patients with stroke. In this sense, our series reflects a coexistence of neoplastic disease in 5%, which is similar to the one present generally in cerebral ischemic disease [31]. Moreover, we obtained 10.6% of heart disease. The assessment and treatment of patients in the TIA Unit allowed for the diagnosis of heart disease with treatment and even admission in patients who were, at first, not admitted by such department.

Regarding the concurrence of cognitive impairment, we obtained an abnormally low figure (6.2% for a mean age of 81.1), in contrast with the incidence for that age group in the general population (10–20%) [32]. A hypothesis that would justify this, although we cannot prove it, could be the fact that elderly patients consulting for self-limited neurological deficits have a good performance status, whereas the same deficits would be less eloquent and assessed in patients with a greater baseline impairment.

There are also socioeconomic data that endorse TIA units, which are the significant reduction in the risk of fatal stroke or stroke causing dependence (m-Rankin > 2), a reduction of hospital admissions for recurrent strokes, a shorter hospital stay and financial savings for each patient [33,34]. In our study, a saving of 1470 hospital stays is reflected.

## Conclusions

This model of TIA unit allows an early diagnosis and treatment of TIAs, preventing recurrences of stroke in the long term. It enables the detection of unstable patients for their admission, hence a scarce number of atherothrombotic cases, and it reflects the cost-effectiveness of using Holter recording to detect cardioembolic cases, which increases as the study is repeated. This model provides the same quality of management than hospital admission, with a significant savings in hospital stays. Moreover, it shows safety in terms of treatments, without complications derived from them, with the inconvenience of some diagnostic mistakes due to the quick management of the disease, and in spite of the fact that the diagnosis has been carried out by a vascular neurologist.

## Competing interests

All authors declared they have no competing interests.

## Authors’ contributions

LB carried out the patient’s reclutation, the statistically analysis, drafted the manuscript and contributed to the study design. SC contributed to the patient’s reclutation and the study design, and drafted the manuscript. DL contributed to the patient’s reclutation. JV contributed to the patient’s reclutation. GM contributed to the patient’s reclutation. JP allows the study development in the Department he leads. CHL allows the study development in the Departement he lead, and contributed to the study design. All autohors read and approved the final manuscript.
